# Pharmacotherapy of Anxiety Disorders: Current and Emerging Treatment Options

**DOI:** 10.1176/appi.focus.19203

**Published:** 2021-06-17

**Authors:** Amir Garakani, James W. Murrough, Rafael C. Freire, Robyn P. Thom, Kaitlyn Larkin, Frank D. Buono, Dan V. Iosifescu

## Abstract

(Appeared originally in *Frontiers in Psychiatry* 2020 Dec 23; 11:595584)

